# Trans crystallization behavior and strong reinforcement effect of cellulose nanocrystals on reinforced poly(butylene succinate) nanocomposites†

**DOI:** 10.1039/c8ra01868e

**Published:** 2018-04-24

**Authors:** Taeho Kim, Hyeonyeol Jeon, Jonggeon Jegal, Joo Hyun Kim, Hoichang Yang, Jeyoung Park, Dongyeop X. Oh, Sung Yeon Hwang

**Affiliations:** Research Center for Industrial Chemical Biotechnology, Korea Research Institute of Chemical Technology (KRICT) Ulsan 44429 Republic of Korea dongyeop@krict.re.kr jypark@krict.re.kr crew75@krict.re.kr; Green Chemistry and Environmental Biotechnology, University of Science and Technology (UST) Daejeon 34113 Republic of Korea; Department of Polymer Engineering, Pukyong National University Busan 48547 Republic of Korea; Department of Applied Organic Materials Engineering, Inha University Incheon 22212 Korea

## Abstract

Biodegradable poly(butylene succinate) (PBS) nanocomposites are polymerized *via in situ* polymerization of succinic acid (SA) with cellulose nanocrystal (CNC)-loaded 1,4-butanediol (1,4-BD) mixtures. As reinforcement fillers, whisker-like CNCs are first dispersed in alcohol and sequentially spray-dried, before adding them to 1,4-BD. During the polymerization, the remains of sodium sulfonate in the CNC surfaces retard the polycondensation reaction, which is carefully controlled for the CNC-loaded systems. For the 0.1–1.0 wt% CNC-loaded PBS nanocomposites, it is found the nano-fillers are sufficiently dispersed to induce different crystallization behavior of the matrix polymer. The CNCs may initially act as heterogeneous nucleation sites of the molten PBS chains, during melt crystallization. In this case, most of them tend to be pushed out from the growing crystallites, which develop different nanocomposite morphologies with increasing CNC content. Among the resulting nanocomposites, the 0.1 wt% CNC-loaded system shows the highest tensile strength of 65.9 MPa, similar to that of nylon 6, as a representative engineering polymer as well as 2 fold elongation at break compared with Homo PBS. The *in situ* polymerized CNC-loaded PBS nanocomposites are expected to be a 100% biomass material for a virtuous cycle of biorefinery. Moreover, they demonstrate that the CNC-loaded PBS nanocomposite with a low CNC loading content can be used in various commercial applications for pollution abatement.

## Abstract 2

Biodegradable poly(butylene succinate) (PBS) nanocomposites are polymerized *via in situ* polymerization of succinic acid (SA) with cellulose nanocrystal (CNC)-loaded 1,4-butanediol (1,4-BD) mixtures.

## Graphical abstract

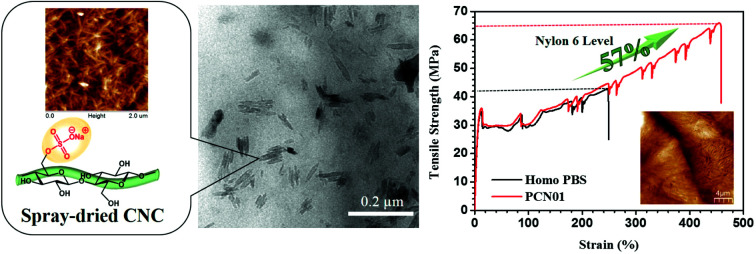

## 1. Introduction

Increasing awareness of global warming and the steady rise in environmental regulatory policies are continuing to increase interest in biodegradable materials.^1,2^ Unlike petrochemical-based materials, however, the application of biodegradable materials has been limited to a few industries owing to the limitation of their physical properties. To overcome these limitations, many researchers have developed biodegradable polymer nanocomposites in the last decade by using various materials (such as clay, layered double hydroxide, carbon nanotubes, graphene oxide) as reinforcement fillers.^3–10^ Recently, many works have been focused on replacing non-degradable fillers in biopolymer nanocomposites with renewable resource fillers to realize a virtuous cycle of biorefinery.^11–19^ Cellulose nanocrystal (CNC) has consistently received much attention as a potential filler for bio-based lightweight composites because of its high aspect ratio, low density, and excellent chemical resistance, as well as good mechanical response to stress.^18–20^ In particular, its Young's and elastic moduli are reported to be over 100 and 143 GPa, respectively, originating from strong intermolecular hydrogen bonds.^21,22^ Nevertheless, strong self-association (self-agglomeration) of CNCs occurs in hydrophobic polymers, because of the abundance of hydroxyl groups on the surface, which makes them difficult to redisperse.

It has been reported that various polymer nanocomposites employing CNCs as eco-friendly natural fillers have excellent physical properties. The ultimate challenge in the fabrication of polymer nanocomposites is to well-disperse the CNCs as a hydrophilic organic filler in a hydrophobic polymer to obtain optimum physical properties. So far, excellent physical properties have been reported for CNC nanocomposites with highly polar polymers such as nylon, polyurethane^18,23,24^ or hydrophilic polyvinyl alcohol (PVA).^11^ However, it is very difficult to disperse the CNCs into a linear biodegradable polymer with very low polarity and high hydrophobicity. In order to overcome this problem, several groups have used solvent casting to prevent self-association behavior or melt compounding process with modified CNCs to lower the hydrophilicity.^16–18,25,26^ Even of these methods are costly, it is difficult to obtain commercial nanocomposites with satisfactory physical properties. Improving the dispersibility of CNCs in a linear biodegradable polymer thus remains a big challenge.

It is reported that the dispersibility of CNCs in a target polymer matrix is significantly affected by preliminary treatments of CNCs, such as dissolving, drying, *etc.*^27^ Based on our preliminary result, which showed the good dispersity of spray-dried CNCs in 1,4-butanediol (1,4-BD), poly(butylene succinate) (PBS) nanocomposites including different CNC loadings were fabricated by *in situ* polymerization of succinic acid (SA) with CNC-loaded 1,4-BD mixtures. It was found that the remains of sodium sulfonate in the well-dispersed filler surfaces retard the transesterification polymerization, producing different physical properties of the resulting CNC-loaded PBS nanocomposites and we confirmed that the CNCs were randomly and well-dispersed in the PBS matrix. Consequently, we successfully prepared PBS-CNC nanocomposites with highly dispersed CNCs in the matrix, and they exhibited far better mechanical and thermal properties than Homo-PBS despite the use of a very low concentration of CNCs (0.1 wt%) as the reinforcement filler. Theses result suggest that PBS-CNC nanocomposites with 100% biomass content can contribute to the biorefinery virtuous cycle in various commercial applications such as fishing gear, coatings, sheets, architecture, ropes, and injection molding for automotive materials.

## 2. Experimental

### 2.1 Materials

CNCs were purchased from Process Development Center of the University of Maine, USA. 1,4-Butanediol (1,4-BD, ≥99%), succinic acid (SA, ≥99%) and titanium(iv) butoxide (as a catalyst) were purchased from Sigma-Aldrich. All reagents were high-purity commercial products and used as received.

### 2.2 Characterization

The number-average molecular weight (*M*_n_), weight-average molecular weight (*M*_w_), and polydispersity index (PDI = *M*_w_/*M*_n_) of each PBS were determined by gel permeation chromatography (GPC, ACQUITY APC XT, Waters Corp.), which equipped an refractive index detector (ACQUITY, Waters Corp.), with chloroform (CF) as an eluent. The intrinsic viscosity of different CNC-loaded PBS nanocomposites dissolved in CF (5 : 5 in volume) was measured using an Ubbelohde viscometer at 25 ± 0.1 °C. In addition, the moisture contents analysis were conducted using a Karl-Fisher systems under an argon-purged glovebox (O_2_ < 0.1 ppm and H_2_O < 0.1 ppm). The water content of the PBS with CNC composites were measured by Karl-Fischer titrator equipped with a 917 Coulometer and 885 Compact Oven (Metrohm, Switzerland). The quantitative moisture were calculated by coulometric method in the saturation regime using Karl-Fisher titration solution (HYDRADAL, Honeywell, Germany), and the values were reported below Table 3.

The dispersion test for CNC which dried various method was conducted in 1,4-BD (1.0 wt%). An ultrasonication was applied for 5 min in water bath (25 °C).

The thermal properties of these samples were investigated using differential scanning calorimetry (DSC, Q-2000, TA instruments) with both heating and cooling rates of 10 °C min^−1^. Thermogravimetric analysis (TGA, Pyris 1, Perkin-Elmer) was conducted to determine the decomposition temperature at 5% weight loss. Also, rheological responses of all the samples were monitored using a dynamic oscillatory viscometer (MCR 302, Anton Paar), where the disk-type specimens with a diameter of 25 mm were placed between the parallel plates with a gap of 1.0 mm; the oscillation frequency sweep test was conducted at 150 °C with a range from 0.1 to 500 rad s^−1^ and 10% strain. The dynamic mechanical properties of PBS and the CNC-loaded nanocomposites were investigated using a dynamic mechanical analyzer (DMA8000, Perkin-Elmer) under an air atmosphere; the specimens were deformed with a tensile mode (frequency of 5 Hz and 0.02% strain).

Tensile test specimens of CNC-loaded PBS nanocomposites were prepared by hot-pressing at 150 °C for 5 min with 100 bar pressure. The dimension of samples were dog-bone shape (63 × 26 × 1.0 mm^3^ as length, width and thickness). Mechanical properties were measured by universal testing machine (UTM, Instron 5943, UK) with a speed of 10 mm min^−1^ at 1 kN load cell.

The dispersion of pristine CNC (spray-dried type in this study) was confirmed by transmission electron microscopy (TEM). CNC solution prepared with 0.1 wt% in water and dropped onto carbon-coated copper grids. These prepared samples dried on the hot plate at 40 °C (1 h) before observation.

Also, CNC-loaded PBS nanocomposite specimens for TEM (70 nm) were prepared by cryo-microtoming (EM UC7, Leica) at −60 °C which transferred onto carbon coated Copper Grids (TED Pella Inc., 200 Mesh Copper Grid). For observing of the cellulose nanocrystal (CNC) clearly, the sectioned films were stained by 1% of osmium tetroxide (OsO_4_) solution vapor for 90 min. Then the prepared films were rinsed with DI water and dried. The morphologies of the CNC-loaded PBS nanocomposite films were confirmed using high resolution TEM (HR-TEM, JEOL, JEM-2100F with Cs corrector, 200 kV). The spherulitic crystallization morphology of PCN films were prepared on the silicon wafer as a substrate. The morphology, size, and thickness of the PCN films were characterized by atomic force microscopy (AFM, Multimode 8, Bruker) using a tapping-mode. A polarizing optical microscope (POM) with CCD camera (Olympus BX51TF) was used to observe the spherulitic growth morphology in PBS and CNC-loaded PBS. A 2 mg sample was placed between two glass slides and melted on a hot plate at 150 °C for 5 min. The molten film was quickly moved to a heating stage (FP82HT, Mettler-Toledo, Greifensee, Switzerland) and held at the crystallization temperature of 90 °C.

### 2.3 Synthesis of PBSs and sample preparation

CNC-loaded PBS nanocomposites were prepared by a two-step melt-condensation process. The CNCs (0.1–1.0 wt%) were dispersed in 1,4-BD by an ultrasonicator (VCX500, 500 W, 20 kHz; Sonic, Danbury, CT, USA) for 5 min. Before the esterification, the flat bottom glass reactor equipped mechanical stirrer, condenser, temperature sensor and N_2_ inlet was purged with nitrogen for 3 h to prevent the oxidation reaction by an oxygen residue. Then, SA was added to the different CNC-loaded 1,4-BD mixtures; for the CNC-loaded mixtures, a molar ratio of [COOH]/[OH] was fixed to 1.2. The mixtures were heated to 150 °C with a rate of 2–3 °C min^−1^ under an N_2_-purged condition. Titanium(iv) butoxide (150 ppm in total weight of monomers) was transferred into the mixtures. The customized round bottom glass reactor equipped with torque measuring mechanical stirrer and an outlet connected to the vacuum pump was heated to 200 °C with a rate of 2–3 °C min^−1^, and the polycondensation time was adjusted to yield similar degree of polymerization of PBS. During the reaction, the products were separated by the removal of water; in this case, temperature was then slowly increased to 240 °C while the stirring speed was maintained at 150 rpm. In addition, the vacuum was gradually reduced below 100 mTorr to effectively extract by-products and the stirring speed was slowly decreased to 30 rpm with an increase in degree of polymerization. The final products were removed from the reactor, sequentially quenched in a water bath, and dried in vacuum oven for 24 h at 80 °C.

The CNC-loaded PBS nanocomposites are denoted by their CNC content; for example, PCN01 represents the CNC-loaded PBS nanocomposite with 0.1 wt% of CNCs.

## 3. Results and discussion

### 3.1 Cellulose-based reinforcement fillers and their dispersion

**Scheme 1:**
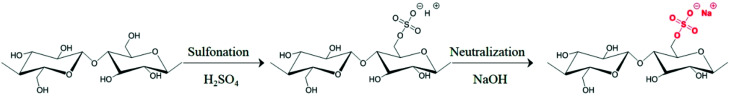
The preparation method of CNC using sulfuric acid.

The CNCs were produced from various cellulose sources such as wood pulp and lint by sulfuric acid hydrolysis (Scheme 1). The unique properties of the CNCs strictly depended on both physical factors such as crystal dimensions, aspect ratio, and surface area and chemical factors such as sodium sulfate concentration and hydrophilicity.^28–31^ Thus, it is important to characterize physical and chemical properties of the CNC-based fillers, to reinforce the polymer matrix.

**Fig. 1:**
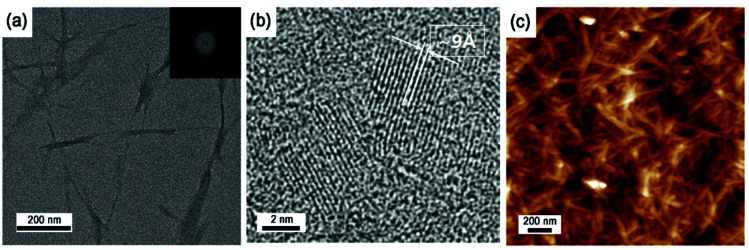
Morphology of CNCs: (a and b) TEM, (c) AFM images.

Fig. 1 shows TEM micrographies of spray-dried CNC mounted on a Cu grid. The spray-dried CNCs showed whisker-like aggregates, which had average width of 20 nm and lengths ranging from 200–400 nm. The CNCs consisted of multi-stacked crystals with a layer spacing of approximately 9 Å due to strong hydrogen bonding and cross linkage between nanocrystals.^26,32,33^ Since typical CNCs are produced *via* hydrolysis and neutralization procedures of various cellulose sources (Scheme 1), the sodium sulfonate moieties are always present on the CNC surfaces, generating considerable charges on the surface.^29,32^ Sodium sulfonate content in the spray-dried CNC was indicated as approximately 0.183 mmol g^−1^, as determined by a titration method (Fig. S1 in ESI†).^31^ The sodium sulfonate effects on the *in situ* polymerization of the CNC-loaded series will be discussed later.

**Fig. 2:**
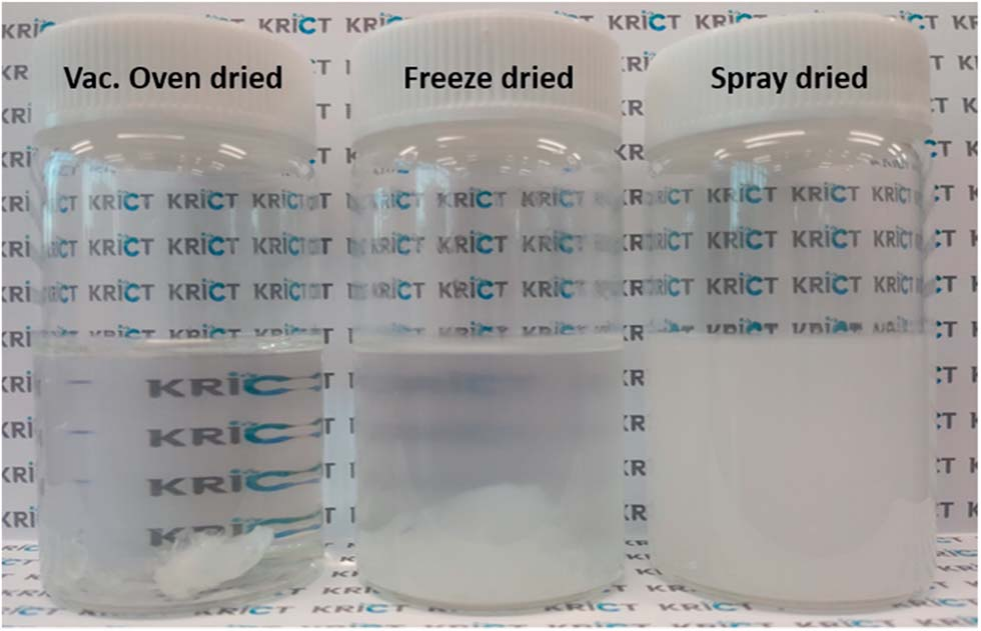
The image of three different types of 1.0 wt% CNC solutions dispersed in 1,4-BD after sonication for 5 min.

It is challenging to fully disperse hydrophilic cellulose-based nanofillers in the PBS matrix as a hydrophobic polymer. Here, CNC-loaded PBS nanocomposite series was fabricated *via* an *in situ* polymerization of SA with different filler-loaded 1,4-BD mixtures. Note that homogeneous dispersion of CNCs in 1,4-BD was an essential step to obtain excellent physical properties of the resulting PBS nanocomposites. To investigate the dispersibility of three different types of nanofillers in 1,4-BD, first, each mixture with 1 wt% filler loading was prepared *via* ultrasonicating for 5 min. As expected, the cellulose-based nanofillers showed different dispersibility in 1,4-BD. As shown in Fig. 2, spray-dried CNC could be sufficiently dispersed in 1,4-BD without any aggregate, in comparison to others presenting precipitates or macro aggregates. Based on the result, the spray-dried CNC was selected as the reinforcement filler for the *in situ* polymerized PBS nanocomposites.

### 3.2 *In situ* polymerization of different CNC-loaded PBS nanocomposites

As mentioned in the Experimental section, PBS and different CNC-loaded nanocomposite series were polymerized. The typical characteristics of the resulting samples are summarized in Table 1. Based on *M*_n_, *M*_w_, and PDI values determined by GPC, it was found that all the PBSs synthesized had the degree of polymerization (DP) greater than 170, similar to those of commercial polymer grades. However, the polycondensation time of the CNC-loaded nanocomposite series drastically increased from about 2 h to 16 h 30 min, with an increase in CNC loading contents. During trans esterification, the primary alcohol in 1,4-BD could attack carbonyl carbon atoms in SA, generating bis(4-hydroxyl butyl) succinate (BHBS) with DP < 10. The presence of sodium sulfonate moieties might cause a steric hindrance adjacent to the carbonyl carbon of SA, increasing the portion of unstable BHBS during the polymerization. As a result, higher CNC loading leads to longer polymerization time.

**Table 1:** Characteristic properties of PCN samples polymerized with different CNC loadings

Sample code^a^	*η*_inh_ b	*M*_n_ (g mol^−1^)	*M*_w_ (g mol^−1^)	PDI	*T*_g_ c (°C)	*T*_c_ d (°C)	*T*_m_ d (°C)	PC time^e^
Homo PBS	0.86	37 500	69 000	1.84	−15.1	72.9	114.2	2 h 20 min
PCN01	0.89	42 200	82 000	1.94	−13.7	73.3	114.0	2 h 15 min
PCN03	0.91	46 400	84 000	1.81	−13.1	77.1	114.0	3 h 30 min
PCN05	0.84	32 100	60 500	1.88	−12.4	80.0	114.0	6 h 35 min
PCN10	0.81	30 000	51 300	1.71	−7.1	84.4	113.8	16 h 30 min

a The number at the end of each samples code denotes the CNC contents. For example, PCN01 represents CNC with 0.1 wt% contents in PBS matrix.

b 0.5 g dL^−1^ in chloroform at 30 °C.

c Measured by DMA, 10 °C min^−1^.

d Measured by DSC, 10 °C min^−1^.

e PC: polycondensation time.

**Fig. 3:**
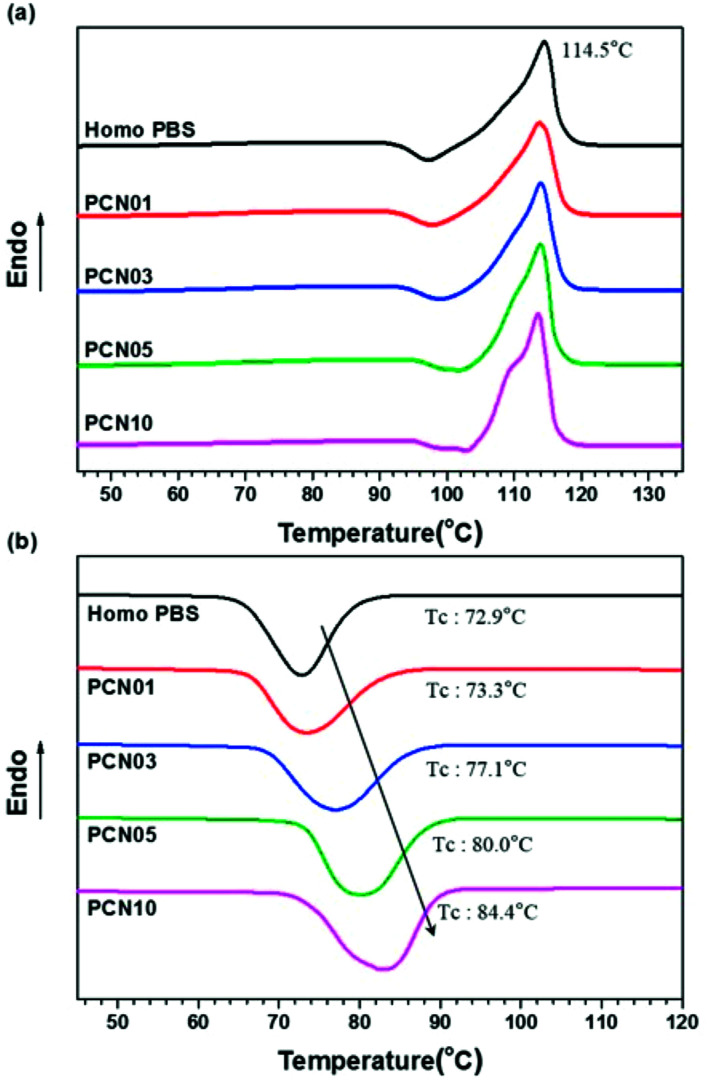
DSC thermograms of Homo PBS and PCN samples: (a) heating and (b) cooling curves with a constant rate of 10 °C min^−1^.

**Fig. 4:**
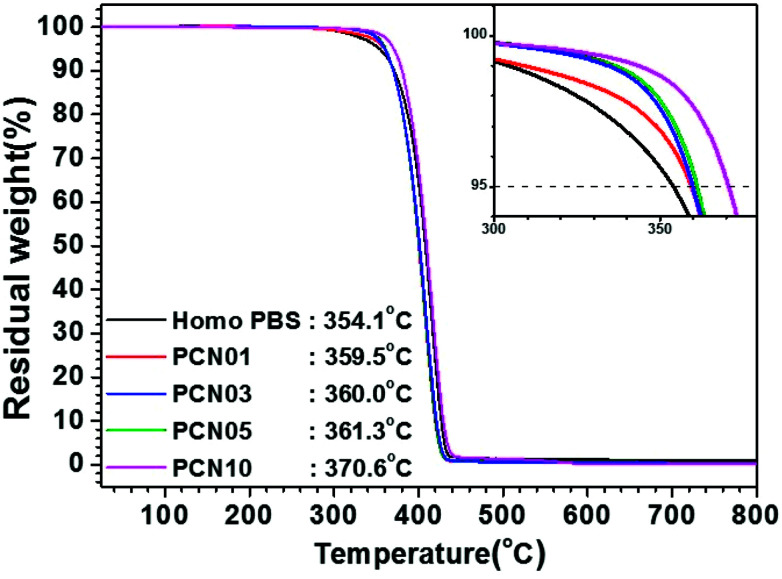
TGA curves of Homo-PBS and PCN samples with different CNC loadings.

As shown in Table 1 and Fig. 3, melting peaks (*T*_m_) of the semi-crystalline PBS synthesized were indicated at temperatures ranging from 114.0 to 114.5 °C in DSC heating curves, independent of the presence of CNCs. Interestingly, crystallization peaks (*T*_c_) of PBS from the molten state increased from 72.9 to 84.4 °C in DSC cooling curves with increasing the CNC loading. The results strongly support that the CNCs dispersed in the PBS matrix act as nucleation sites and the presence of the CNCs does not disturb the crystal growth (propagation) of PBS, as well as crystal packing; less-ordered and small-sized crystallites tend to be molten at lower temperatures, in comparison to ordered and large-sized ones.^34–38^ In addition, the glass-transition temperature (*T*_g_) for these samples determined by DMA increased from −15.1 to −7.1 °C with increasing the CNC content, suggesting that the mobility of the PBS backbone is limited by strong affinity of the chain to the surfaces of well-dispersed CNCs. In particular, the presence of the CNCs with high endothermic heat capacity enhanced the thermal stability of the CNC-loaded nanocomposites. Fig. 4 represents TGA curves of the different CNC-loaded nanocomposites. For the PBS itself, the value of decomposition temperature (TD) known as a temperature at 5% weight loss, 354.1 °C, while for the CNC-loaded nanocomposites the values increased gradually from 359.5 to 370.6 °C with an increase in the CNC loading contents.

### 3.3 Mechanical properties of PBS and CNC-loaded nanocomposites

To investigate the effect of the CNCs as a reinforcement filler, dumbbell-shaped specimens of the PBS and CNC-loaded nanocomposites were mechanically tested (Fig. S2 in ESI†) and the resulting properties are summarized in Table 2. First, average tensile strengths of the CNC-loaded nanocomposites decreased monotonically from 65.9 to 40.9 MPa with an increasing in the CNC loading. Among the nanocomposites, the 0.1 wt% CNC-loaded system showed the maximum value of 65.9 MPa, which was 157% of that obtained for the PBS itself and comparable to those of nylon 6 ones reported in elsewhere.^39,40^ In particular, its elongation at break was twice that (230 ± 20%) of the PBS system. The results are mainly related to the sufficient dispersion of CNCs in the PBS matrix *via in situ* polymerization of SA with hydrophilic CNC-loaded 1,4-BD mixtures. In addition, the strong hydrogen bonding of carbonyl groups of PBS chains with the hydroxyl moieties on the CNC surfaces enhanced the mechanical properties.^19,23^

**Table 2:** Mechanical properties of Homo PBS and PCN samples

Sample^a^	Tensile strength (MPa)	Young's modulus (MPa)	Elongation at break (%)	Toughness (MPa)
Homo-PBS	42.1 (0.7)	688.5 (30.2)	230 (20)	88.9 (4.8)
PCN01	65.9 (2.2)	682.3 (16.3)	450 (20)	200.2 (9.2)
PCN03	61.1 (0.7)	751.1 (8.6)	275 (1)	181.8 (2.3)
PCN05	54.3 (1.2)	826.2 (11.8)	357 (15)	140.0 (6.8)
PCN10	40.9 (1.3)	898.6 (20.3)	8 (1)	2.19 (5.7)

a Values in parentheses are standard deviations.

**Fig. 5:**
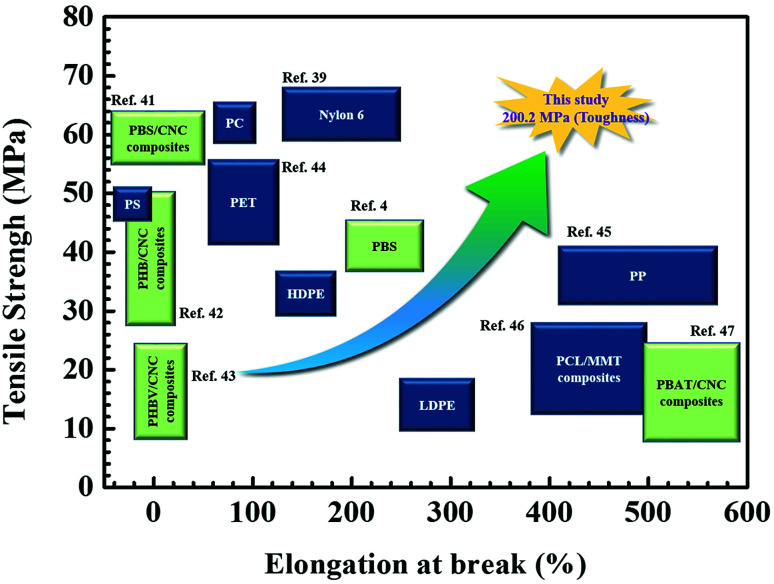
Schematic diagram of mechanical properties of the 0.1 wt% CNC-loaded nanocomposites compared with biodegradable polymer, their nanocomposites and petrochemical polymer.^4,39,41–47^

Since Young's modulus (*E*) values of CNCs are extraordinary high as much as 105–143 GPa,^28^ it was expected that the CNC-loaded nanocomposite series was expected to have a drastic enhancement in *E*. However, the resulting *E* values of the nanocomposites increased monotonically from 682.3 ± 16.3 (for 0.1 wt% CNC loading) to 898.6 ± 20.3 MPa (for 1.0 wt% CNC loading). With the benefit of both the improved tensile strength and elongation at break, the PBS nanocomposites, except for 1 wt% CNC loading, yielded a drastic increase in toughness up to approximately 200 MPa, in comparison to 88.9 ± 4.8 MPa of the PBS only. For most of inorganic nanofiller-loaded polymer nanocomposites, at least 1% inorganic particles are necessary to improve the mechanical properties.^7,8^ However, the result is encouraging that the small amount of CNCs (<1 wt%) loaded in the PBS *via* the *in situ* polymerization could provide a tremendous improvement in physical properties. In particular, it is very rare for the tensile strength, toughness, and modulus of a nanocomposite to be enhanced at the same time. As shown Fig. 5, this result, which the maximum value of 200.2 MPa for toughness, shows better physical properties than the various biodegradable polymers and their composites as well as the representative petrochemical polymers (Movie S1 in ESI†).

### 3.4 The resulting crystalline structures

**Fig. 6:**
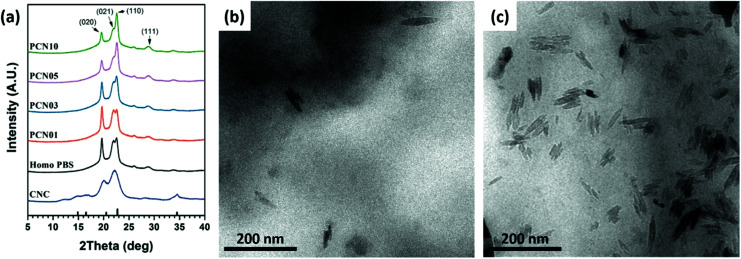
(a) WAXD patterns of CNCs and the PCN samples. TEM micrographs of (b) PCN03 and (c) PCN10.

Wide-angle X-ray diffraction (WAXD) was performed on the CNC, PBS, and CNC-loaded nanocomposites to investigate the nanofiller effects on the crystalline structures of the PBS matrix. First, typical X-ray diffraction pattern of the spray-dried CNC powder showed reflection peaks at 2*θ* = 14.8, 16.5, 22.2 and 34.5°, originating from a cellulose I structure (Fig. 6a).^16,48^ In addition, PBS film showed reflection peaks at 2*θ* = 19.6, 21.9, 22.7, and 28.8°, corresponding to (020), (021), (110), and (111), respectively.^49,50^ Note that the X-ray reflection peaks at 20.1 and 28.2° from the CNC crystals are overlapped with some crystal reflections of PBS and its nanocomposites, making it difficult to confirm the degree of dispersion of the CNCs. However, since there was no change in intensity change for the peaks at 14.8° and 34.5°, it can be concluded that the CNCs were well dispersed in the PBS matrix. This result indicates that 1.0 wt% of CNCs could be effectively dispersed. As the CNC content increased, the intensity of the (110) peak increased and that of the (020) peak decreases, indicating that the CNCs were a factor in the PBS crystal growth structure. The WAXD patterns provided no direct information on the degree of dispersion of CNCs.

Therefore, TEM was conducted on the CNC-loaded nanocomposites to observe the degree of the dispersion of the CNCs in the PBS matrix. Fig. 6 represents TEM micrographies of different CNC-loaded nanocomposites showing well-dispersed CNCs in the matrix. For all the nanocomposites, CNCs were well-dispersed in the matrix with whisker-like nanodomains (width ∼ 100 nm, length = 10–20 nm).

**Fig. 7:**
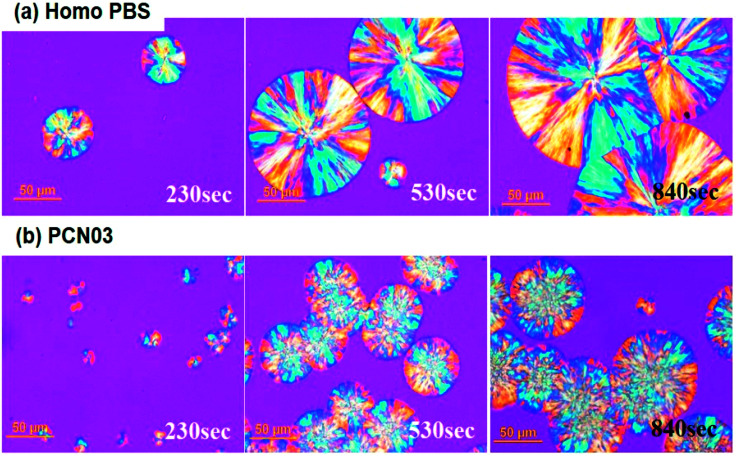
Polarizing optical microscope images of (a) Homo-PBS and (b) PCN03 during isothermal crystallization at 90 °C.

Overall crystal morphologies of the different CNC-loaded PBS nanocomposites were investigated using both polarized optical microscopy (POM) and AFM. 100 μm-thick samples were completely molten at 150 °C, and then the crystal growth of PBS were monitored *via* isothermally crystallizing at 90 °C. Similar to most semicrystalline polymers, PBS started to develop lamellae at primary nucleation sites and radially grew into spherulitic crystals (left side in Fig. 7a), which showed typical maltese cross patterns with a negative birefringence character under a polarized light.^51,52^ As the isothermal crystallization time (*t*_iso_) increased, the growing spherulites be collided with the nearest neighbor (middle side in Fig. 7a), and the completely-crystallized film showed clear grain boundaries (GBs) between the spherulites with average diameter of 150–200 μm (right side in Fig. 7a). In case of the CNC-loaded PBS systems, the polymer matrix could be quickly crystallized from much larger number of nucleation sites than those in the no filler system; the higher filler content induced the higher nucleation sites. Interestingly, the spherulites grown inside the nanocomposites showed different morphologies in POM images, including the dispersed CNCs increasing the crystallization kinetics the spherulite morphology was different from that of Homo-PBS: the spherulite size of PCN03 was lower and its density was higher because of the large amount of CNCs as nucleating sites (Fig. 7b). As shown in Fig. 7c, the average rate of spherulite growth in the PCN samples was relatively smaller than that in Homo-PBS because of its high nuclei density. This result offers conclusive evidence that the CNCs could act as a nucleating agent in the PBS matrix. However, the analysis of growth morphology of spherulites, in which the interface was not formed and collapsed, must take into consideration that the CNCs could show different types of crystallization behavior during isothermal crystallization. Most of the studies in the literature reported that semicrystalline polymer or fiber–filler-reinforced composites exhibit induced transcrystallization behavior, with the transverse fiber surfaces nucleating more than the longitudinal surfaces.^53–55^

**Fig. 8:**
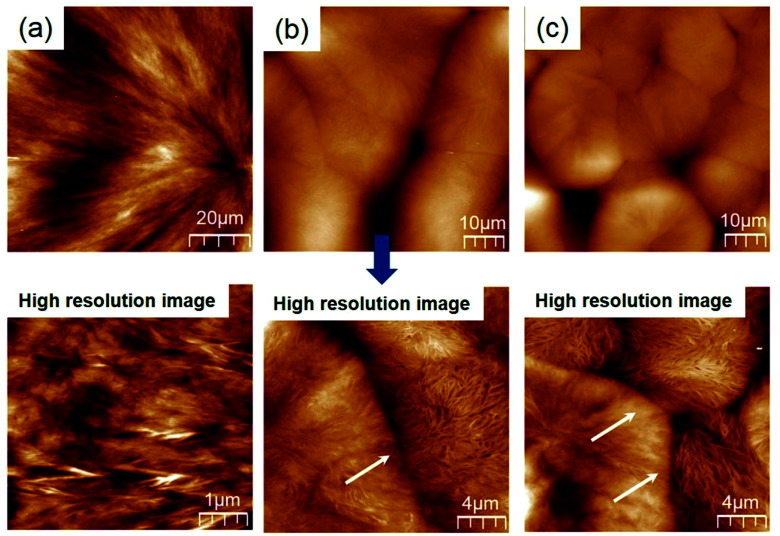
High-resolution AFM topographies of spherulites containing different CNC loadings: (a) Homo-PBS; (b) PCN03; (c) PCN 05 (at a high resolution images).

The AFM topographies were measured to observe the microstructure at the spherulitic interface. As shown Fig. 8a, Homo-PBS had a fibrous structure and a clean maltese cross in the spherulites, which grew from the core along the radial direction. The spherulitic interface of the PCN samples was not formed and collapsed, as shown Fig. 8b and c, which is consistent with the POM images. However, particularly distinguishing morphologies such as intestinal villi were observed in the vertical direction of the interface in the high-resolution images, which can be explained by the CNCs being pushed out of the core and inducing transcrystallization *via* heterogeneous nucleation.^53–55^

### 3.5 Rheological properties

**Fig. 9:**
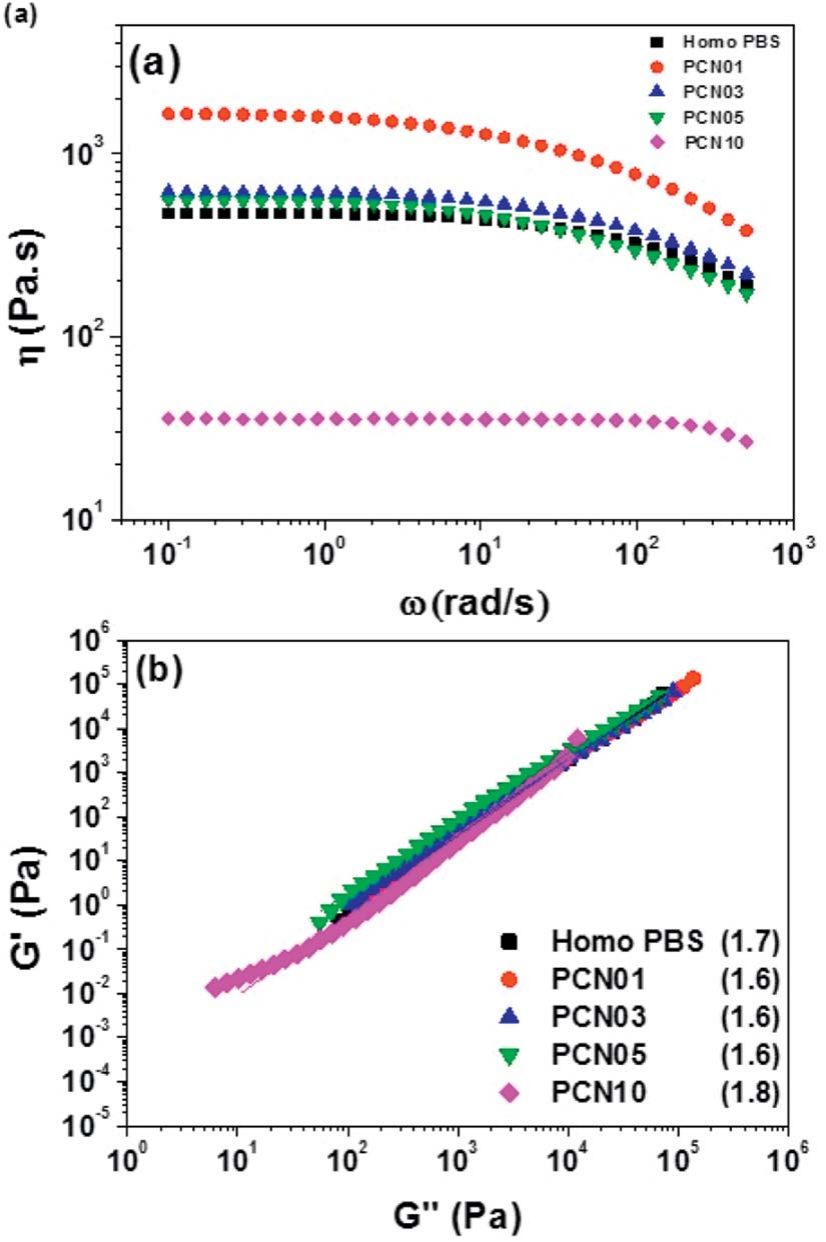
(a) Master curves of dynamic melt viscosity and (b) Cole–Cole plots of the PCN series; the values in parentheses are the slopes of the Cole–Cole plots.

It is important to investigate the rheological properties of polymers used for various industrial processes. Therefore, the viscoelastic response of the PBS nanocomposite series in a molten state was investigated using rheometry. Fig. 9 represents the shear viscosity of Homo-PBS and PCN01 indicate the presence of a Newtonian fluid in regions of low shear rate. These regions exhibited shear-thinning behavior with increasing shear rate. However, the shear viscosity of PCN01 increased relative to that of Homo-PBS in the shear ranges, indicating that the CNCs were fully dispersed in the PBS matrix, which substantially enhanced the interfacial interaction between functional molecules of the CNCs and PBS matrix. In general, the shear viscosity of an inorganic nanocomposite increases with increasing inorganic content at low shear owing to the restriction of polymer chains and sometimes exhibit higher degrees of shear-thinning behavior compared to monomers such as Bingham fluids.^56,57^ The viscosity of the PCN series gradually decreased with increasing CNC content after the viscosity peaked in PCN01.

**Fig. 10:**
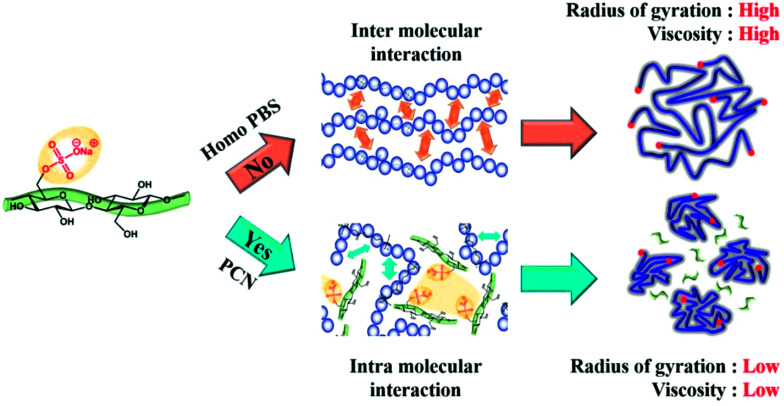
Schematic diagram of the effect of CNCs on the change in interaction of the PBS backbone.

The water content of the CNCs and the PCN samples was measured to determine the main factors that decreased the viscosity of the PCN series according to the CNC content. As shown in Table 3, the water content of the CNCs was 4500 ppm. However, the concentration of water in the prepared PCN nanocomposites was only 3–4 ppm, which is similar to that in Homo-PBS. This means that the residual water content was removed with the byproduct during polymerization, indicating that the water content was not the cause of the decrease in viscosity. As mentioned earlier, we anticipated that the amount of sulfonate groups in the CNCs (0.183 mmol g^−1^) would affect the viscosity of the PCN nanocomposites as well as the polymerization time, as indicated by the amount of sodium sulfonate on the remaining functional group of CNCs during the neutralization process. As shown in Fig. 10, the increase in the amount of sodium sulfonate on the CNC surface reduced the intermolecular interactions between the CNCs and PBS chains, while increased the intermolecular interactions between ionic groups of CNCs, which caused the reduction in the radius of gyration and chain slip for PBS main chains.^38^ Therefore, the zero-shear viscosity of the PCN composites was lowered as the sodium sulfonate concentration in the CNC particles increased. The Cole–Cole plot of PCN samples show similar slopes of 1.7 for the master curves of samples without CNC (Fig. 9b). In the case of an isotropic and homogeneous polymer, the slope is 2.0 regardless of the temperature range. The slopes of the lines representing PCN samples are similar to that of the line representing Homo-PBS, indicating that the PCN samples were close to a homogeneous system, demonstrating that the CNCs had strong interaction with the PBS backbone and were highly dispersed.

**Table 3:** Water content of CNCs, Homo-PBS and PCN series

Sample	CNC	Dried CNC	Homo-PBS	PCN01	PCN03	PCN05	PCN10
Water content (ppm)	9900 (80)	4500 (130)	3 (0.7)	4 (0.7)	4 (0.6)	3 (0.6)	3 (0.7)

## 4. Conclusions

Nanocomposites of biodegradable aliphatic polyester, PBS, and CNCs with excellent physical and thermal properties were prepared by *in situ* polymerization. To enhancement the dispersion of CNCs in the matrix, spray-dried CNCs were used during polymerization, and the CNCs were preferentially dispersed in 1,4-butanediol. The dispersibility of the CNCs in the PBS matrix samples was confirmed by high-resolution transmission electron microscope micrographs. In the case of sample PCN01, which had good dispersion states, the shear viscosity was higher than that of Homo-PBS over a broad range of shear rates. As a result of the high dispersion of CNCs, the PCN samples not only showed excellent thermal properties, but also a remarkable increase in physical properties such as mechanical and rheological properties despite the addition of only a small amount of CNCs. When the CNCs were homogeneously dispersed in the PBS matrix, it was possible to obtain a strong reinforcing effect even if a very small amount was added. Because of the outstanding results of the PCN samples, we believe organic–organic nanocomposites should replace inorganic nanocomposites reinforced with nanoclay or talc as engineering plastics. These biodegradable organic–organic nanocomposites are also more promising packaging materials that are safe for the environment.

## Conflicts of interest

There are no conflicts to declare.

## Supplementary Material

RA-008-C8RA01868E-s001
