# Correction: Trans crystallization behavior and strong reinforcement effect of cellulose nanocrystals on reinforced poly(butylene succinate) nanocomposites

**DOI:** 10.1039/c8ra90050g

**Published:** 2018-06-13

**Authors:** Taeho Kim, Hyeonyeol Jeon, Jonggeon Jegal, Joo Hyun Kim, Hoichang Yang, Jeyoung Park, Dongyeop X. Oh, Sung Yeon Hwang

**Affiliations:** Research Center for Industrial Chemical Biotechnology, Korea Research Institute of Chemical Technology (KRICT) Ulsan 44429 Republic of Korea dongyeop@krict.re.kr; Green Chemistry and Environmental Biotechnology, University of Science and Technology (UST) Daejeon 34113 Republic of Korea; Department of Polymer Engineering, Pukyong National University Busan 48547 Republic of Korea; Department of Applied Organic Materials Engineering, Inha University Incheon 22212 Korea

## Abstract

Correction for ‘Trans crystallization behavior and strong reinforcement effect of cellulose nanocrystals on reinforced poly(butylene succinate) nanocomposites’ by Taeho Kim *et al.*, *RSC Adv.*, 2018, **8**, 15389–15398.

Fig. 1(b) shown in the published article was incorrect and the revised figure and legend are shown below. In addition, a sentence of text “The CNCs consisted of multi-stacked crystals with a layer spacing of approximately 9 Å due to strong hydrogen bonding and cross linkage between nanocrystals” should be removed from the second paragraph of the Results and discussion section.

**Fig. 1:**
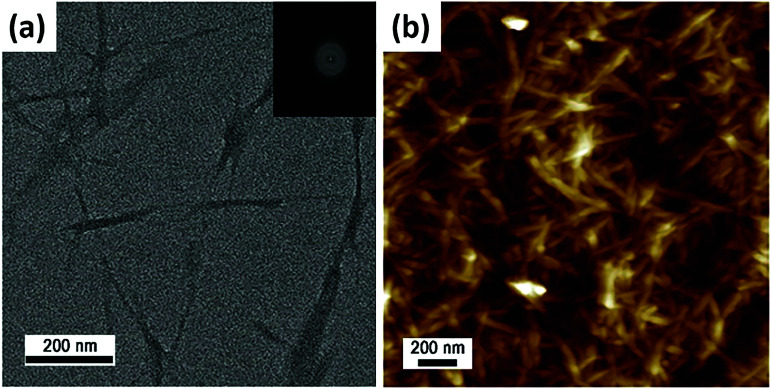
Morphology of CNCs: (a) TEM, (b) AFM images.

The Royal Society of Chemistry apologises for these errors and any consequent inconvenience to authors and readers.

## Supplementary Material

