# Supplementary material for: Trans crystallization behavior and strong reinforcement effect of cellulose nanocrystals on reinforced poly(butylene succinate) nanocomposites
Source: RSC Adv. 2018 Apr 24;8(28):15389–98. doi: 10.1039/c8ra01868e (PMC9080038; doi:10.1039/c8ra01868e)
Supplement: RA-008-C8RA01868E-s001 [file RA-008-C8RA01868E-s001.pdf]

Electronic Supplementary Material (ESI) for RSC Advances.
This journal is © The Royal Society of Chemistry 2018

Supporting Information

Trans crystallization Behaviors and Strong reinforcement effect of Cellulose nanocrystals on Reinforced Poly (butylene succinate) nanocomposites

Taeho Kim[a,c], Hyeonyeol Jeon[a], Jonggeon Jegal[a], Joo Hyun Kim[c], Hoichang Yang[d], Jeyoung Park[***a,b], Dongyeop X. Oh[**a,b] and Sung Yeon Hwang[*a,b]

[a] *Research Center for Industrial Chemical Biotechnology, Korea Research Institute of Chemical Technology (KRICT), Ulsan 44429, Republic of Korea.*

[b] *Green Chemistry and Environmental Biotechnology, University of Science and Technology (UST), Daejeon 34113, Republic of Korea*

[c] *Department of Chemistry, Pukyong National University, Busan, 48513, Republic of Korea*

[d] *Department of Applied Organic Materials Engineering, Inha University, Incheon 22212, Korea*

To whom correspondence should be addressed E-mail : ***jypark@krict.re.kr (J. Park), **dongyeop@krict.re.kr (D.X. Oh), *crew75@krict.re.kr (S.Y. Hwang).

* Corresponding author.

** Corresponding author.

*** Corresponding author.

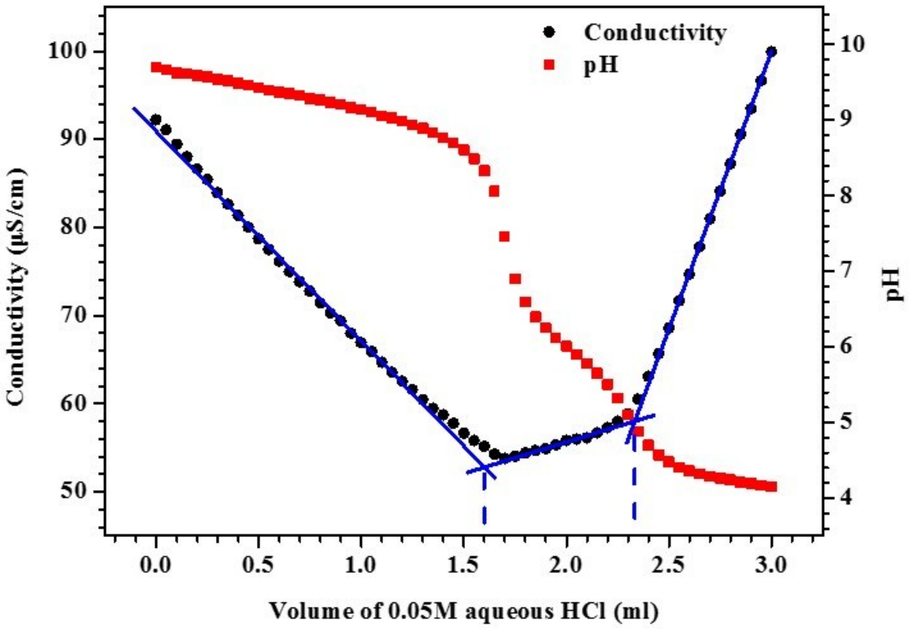

Figure 1S. Activated Na^+ Activated Na^+ cation of CNC by Conductometric titration

0.2g of spray-dried CNC powder was containing in 300ml of Ultrapure (milliQ) water and dispersed by ultrasonication. A pH value of the CNC suspension was initialized by 2M NaOH(aq.) until pH 10. Activated Na^+ cation of CNC was titrated with 0.05M HCl(aq.) step by step. Total amount of added 0.05M HCl(aq.) was 3 ml and the period consist of that 0.05ml of 0.05M HCl(aq.) addition and waiting for 1 min.

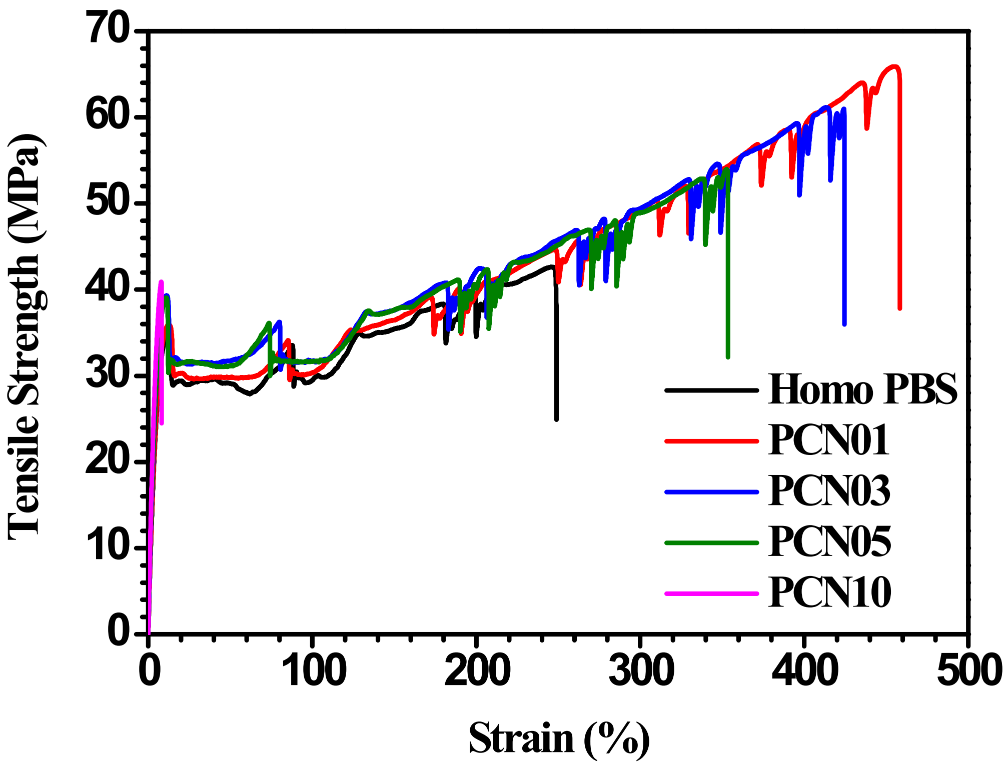

Figure S2. Tensile Properties of Homo PBS and PCN samples: pulling at a rate of 10mm/min

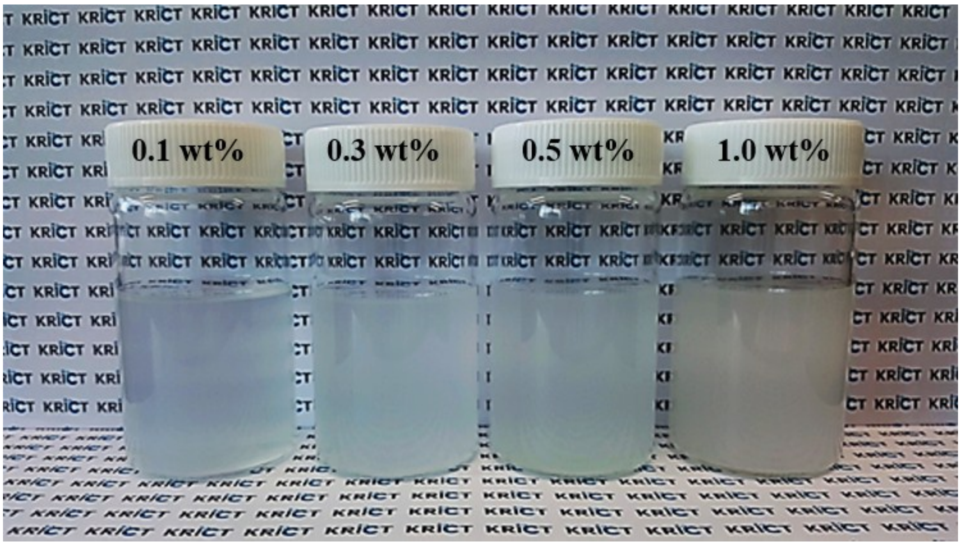

Figure S3. Image of dispersed CNC with increasing concentration in 1,4-butanediol

Movie S1. Mechanical test for PP, Homo PBS and the 0.1 wt.% CNC-loaded nanocoposites
